# Denture stomatitis associated with small-colony variants of *Staphylococcus aureus*: a case report

**DOI:** 10.1186/s12903-019-0910-6

**Published:** 2019-10-11

**Authors:** Katarzyna Garbacz, Ewa Kwapisz, Maria Wierzbowska

**Affiliations:** Department of Oral Microbiology, Faculty of Medicine, Medical University of Gdansk, 25 Dębowa St, 80-204 Gdansk, Poland

**Keywords:** Small-colony variants, *Staphylococcus aureus*, Oral cavity, Denture stomatitis, Gentamicin-resistant

## Abstract

**Background:**

The small-colony variants (SCVs) of *Staphylococcus aureus* were isolated from persistent and recurrent infections, especially after placement of medical devices having direct contact with human tissues. The emergence of SCVs is a survival strategy of *S. aureus* which enables them to hide inside host’s cells and induces a less severe immune response than to wild-type *S. aureus*. However, contrary to other medical devices, dental prosthesis as a surface potentially colonized by SCVs of *S. aureus* has not been examined thus far. We reported the first case of SCVs - *S. aureus* infection in denture wearer.

**Case presentation:**

A 62-year-old woman with a complete removable acrylic denture presented extensive elevated erythematous lesions on the palate, compatible with denture stomatitis. The patient had a history of arterial hypertension, cigarette smoking and wearing denture at night. The fungal colonies, identified as *Candida albicans*, were cultured on Sabouraud agar. From three swabs (from hard palate mucosa, denture surface and angular cheilitis lesions) were cultured of pinpoint, clear, non-pigmented, and non-haemolytic colonies on Columbia agar. The small colonies turned out to be Gram-positive cocci, catalase-, Pastorex Staph Plus -, and clumping factor-positive, and oxidase-negative. Suspected phenotypically SCVs forms were definitively identified as *S. aureus* based on PCR amplification of species specific *nuc* and *coa* genes. Methicillin-resistance was verified by *mecA* gene detection. The isolates turned out to be susceptible to methicillin (MSSA) and resistant to gentamicin. The isolate was identified as menadione-auxotrophic variant.

**Conclusions:**

This case demonstrated that oral cavity in denture wearers may be a reservoir of small-colony variants of *S. aureus,* besides *C. albicans*. The prevalence of these bacteria and their role in the pathogenesis of oral diseases are not understood. Due to problems with their detection and identification, the true prevalence of oral SCVs may be underestimated.

## Background

Denture stomatitis (DS) is an inflammation of oral mucosa beneath dental prosthesis. DS is a very common problem, occurring in more than two thirds of denture wearers. Although this condition may be induced by many factors, one of its most common causes is formation of polymicrobial biofilm on denture surface, which is frequently associated with *Candida* species, particularly *Candida albicans*. Fungal cells easily adhere to acrylic surfaces of denture and support adhesion and colonization by oral bacteria. The polymicrobial denture plaque triggers inflammatory response, which manifests clinically as erythema and hyperplasia. Microorganisms colonizing denture plaque may constitute a risk factor for systemic illness, such as rheumatoid arthritis, cardiovascular diseases, and in particular, respiratory infections. *Staphylococcus aureus* have been isolated from denture plaque and oral mucosa of DS sufferers [1]. However, to the best of our knowledge, small-colony variant (SCV) of *S. aureus* associated with denture stomatitis has not been reported thus far.

## Case presentation

A 62-year-old woman with a complete removable acrylic denture referred to the Denture and Implant Clinic, University Dental Centre at Medical University of Gdansk. She presented with reddening of hard palate mucosa with concomitant burning sensation, discomfort, bad taste and angular cheilitis. Intraoral examination showed presence of extensive elevated erythematous lesions on the palate, compatible with denture stomatitis. The patient had a history of arterial hypertension, cigarette smoking and wearing denture at night. She has neither been hospitalized nor received antibiotics within a year prior to the referral.

**Fig. 1:**
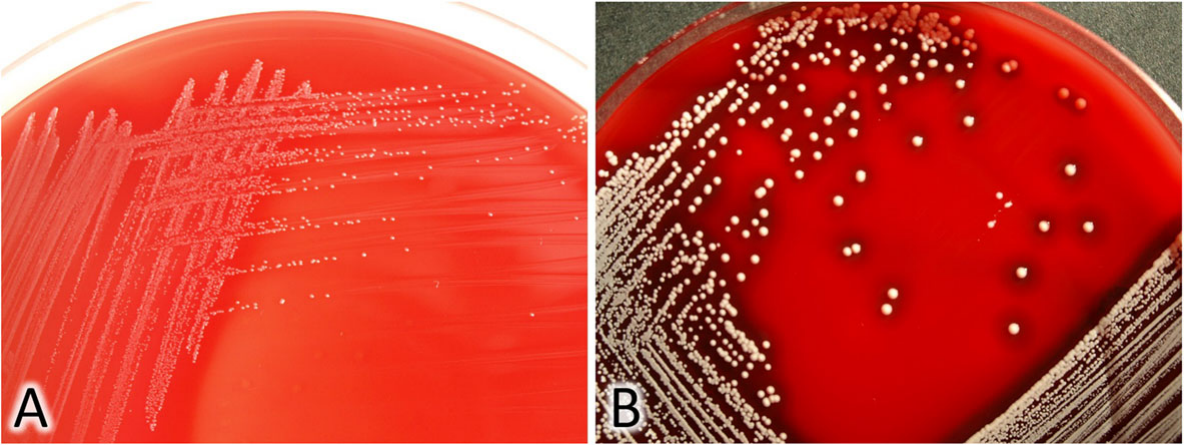
**a,** Blood Columbia agar with colonies of SCVs - *S. aureus* isolated from denture stomatitis. **b,** Blood Columbia agar with normal colonies of *S. aureus* reference strain ATCC25923

Three samples were obtained with sterile cotton swabs: from hard palate mucosa, denture surface and angular cheilitis lesions. The samples were cultured under aerobic conditions on Columbia blood-agar, mannitol salt agar, Sabouraud dextrose agar and MacConkey agar incubated at 35 °C for 48 h. While no growth was observed on MacConkey and mannitol salt agar, yeast-like fungal colonies were cultured from palatal and denture swabs on Sabouraud agar. The fungi were identified as *Candida albicans* based on biochemical testing (API 20C AUX, bioMerieux, Marcy-l’Etoile, France). Furthermore, pinpoint, clear, non-pigmented, and non-haemolytic colonies were recovered on Columbia agar after a 48-h incubation of material from all three swabs (Fig. 1a), in comparison to “normal” colonies of *S. aureus* (Fig. 1b). Gram-stained smears showed presence of Gram-positive cocci arranged in small clusters. The small colonies turned out to be catalase-positive, Pastorex Staph Plus (Bio-Rad, Marnes-la-Coquette, France)- positive, and clumping factor-positive, and oxidase-negative. In the tube coagulase test, a delayed coagulase reaction was observed after a 23-h incubation. The strain was eventually identified as *S. aureus* based on PCR amplification of *nuc* and *coa* genes [2, 3]. Hemin, thymidine and menadione auxotrophy was tested with the disc method. Discs (Sigma-Aldrich, Saint Louis, MO, United States) impregnated with hemin (10 μg/ml), thymidine (100 μg/ml) and menadione (25 μg/ml) were placed on Mueller-Hinton II agar plates inoculated with a 0.5 McFarland standard of the strain. Based on presence of normal-sized, yellow-pigmented colonies around the menadione disc, the isolates were identified as menadione-auxotrophic variant. There were not isolated other “normal” colonies of *S. aureus*.

Antimicrobial susceptibility was determined by disc diffusion on Mueller-Hinton agar supplemented with blood, and interpreted according to the European Committee on Antimicrobial Susceptibility Testing [4]. The panel of tested antibiotics included erythromycin, cefoxitin, clindamycin, ciprofloxacin, tetracycline, gentamicin, sulfamethoxazole/trimethoprim, chloramphenicol and vancomycin (Becton Dickinson, Germany). Gentamicin and vancomycin MICs were determined with E-test (bioMerieux, Marcy-l’Etoile, France). The isolates turned out to be resistant solely to gentamicin (MIC = 16 μg/ml, E-test). Methicillin- resistance was verified by *mecA* gene detection, as described elsewhere [2], with *S. aureus* ATCC 25923 and *S. aureus* ATCC 43300 strains as negative and positive control, respectively. The isolated SCV strain was identified as methicillin-susceptible.

The symptoms of stomatitis resolved after 2 weeks of concurrent therapy with nystatin oral suspension (100 000 IU, once a day in the evening, retained in mouth for 1 min and then swallowed) plus ciprofloxacin (2 × 500 mg) for initial 7 days.

## Discussion and conclusions

Although SCV *S. aureus* have been recently isolated from human and animal infections, as well as from foods [5], to the best of our knowledge, this is the first report documenting identification of such microorganisms in a patient with denture stomatitis. For a long time, isolation of SCVs was believed to be linked to therapeutic or prophylactic use of antibiotics. However, SCVs were recently shown to be a part of normal staphylococcal growth cycle, without any selective antibiotic pressure [6].

SCVs grow slowly on conventional media, forming small, non-pigmented, non-haemolytic colonies. These atypical features are associated with their inability to synthesize thymidine, or with defects in electron transport-chain components, resulting from menadione or hemin auxotrophy. Genetic mutations are responsible for the lack of fermentation of mannitol and other sugars, weak synthesis of species-specific proteins (e.g. coagulase) and lesser susceptibility to antibiotics, such as aminoglycosides, penicillins, trimethoprim/sulfamethoxazole, ciprofloxacin, erythromycin and clindamycin [5, 6].

The SCV strain isolated from our patient was menadione-dependent and resistant to gentamicin. Aminoglycosides are positively charged molecules the transport of which across bacterial membrane is determined by the electrochemical gradient. Resistance of SCVs to aminoglycosides is a consequence of decreased antibiotic uptake, caused by reduced electron gradient across the membrane [7]. Gentamicin-resistant and menadione-dependent SCV strains were isolated from patients with various chronic infections after long-term therapy [5]. In the hereby presented case, the SCV strain emerged although the patient has not been treated with aminoglycosides or other antibiotics. Also other authors reported isolation of SCVs from human infections despite the lack of antibiotic pressure [5, 8]. Edwards [6] showed that gentamicin-resistant SCVs may emerge in the absence of antibiotic pressure by phenotypic switching from the normal phenotype. These staphylococci constitute a permanent reservoir, can persist in host’s tissues and revert to the normal phenotype [6].

The emergence of SCVs is a survival strategy of *S. aureus* which enables them to hide inside host’s cells and induces a less severe immune response than to wild-type *S. aureus* [5]. Therefore, SCVs are an etiologic factor of persistent and recurrent staphylococcal infections, especially after placement of medical devices having direct contact with human tissues. For example, some difficult to diagnose infections were reported after implantation of cardiac pacemakers, insertion of catheters and other devices [9, 10]. Therefore, SCVs are also likely to emerge in denture wearers. However, contrary to other medical devices mentioned above, denture can be easily removed. Nevertheless, our patient declared wearing her denture overnight, which is an established risk factor for DS. Future research should verify whether this also may play a role in the etiopathogenesis of SCV infections.

Recently Bayston et al. [11] reported that the emergence of SCVs may be triggered by triclosan, a synthetic bisphenol antimicrobial agent widely used in hygienic products, such as mouth washes and toothpaste. Perhaps isolation of SCVs from human oral cavity should be analysed also in the context of triclosan-containing product use. To the best of our knowledge, this issue has not been addressed by any previous study.

Aside from SCVs, also *Candida albicans* were isolated from our patient with DS. Published evidence suggests that some dentures may be colonized by up to three different pathogens, among them *C. albicans*. *C. albicans* may form co-aggregates with oral bacteria, including *S. aureus* [1]. The report published recently by Kean also implies that *S. aureus* and *C. albicans* are closely related and interact with each other during the course of polymicrobial infection [12]. Perhaps, like pathogens co-existing with *S. aureus*, e.g. *Pseudomonas aeruginosa*, presence of *C. albicans* may play a role in the emergence of SCVs in the oral cavity. Previous studies demonstrated that co-infection with *P. aeruginosa* may result in SCV selection [13].

In summary, our findings suggest that oral cavity in denture wearers may be a reservoir of small-colony variants of *S. aureus,* besides *C. albicans*. The prevalence of these bacteria and their role in the pathogenesis of oral diseases are not understood. Due to problems with their detection and identification, the true prevalence of oral SCVs may be underestimated.

## Data Availability

Not applicable.

## Abbreviations

API: Analytical profile index
DS: Denture stomatitis
EUCAST: European Committee on Antimicrobial Susceptibility Testing
SCVs: Small-colony variants
